# Staff Perceptions of Preimplementation Barriers and Facilitators to a Mobile Health Antiretroviral Therapy Adherence Counseling Intervention in South Africa: Qualitative Study

**DOI:** 10.2196/23280

**Published:** 2021-04-06

**Authors:** Siobhan McCreesh-Toselli, John Torline, Hetta Gouse, Reuben N Robbins, Claude A Mellins, Robert H Remien, Jessica Rowe, Neshaan Peton, Stephan Rabie, John A Joska

**Affiliations:** 1 HIV Mental Health Research Unit Department of Psychiatry and Mental Health University of Cape Town Observatory South Africa; 2 HIV Center for Clinical and Behavioral Studies New York State Psychiatric Institute and Columbia University New York, NY United States; 3 The Columbia Center for New Media Teaching and Learning Columbia University New York, NY United States; 4 City of Cape Town Metropolitan Municipality Cape Town South Africa

**Keywords:** HIV/AIDS, antiretroviral treatment, low-resource settings, mHealth, Masivukeni, Consolidated Framework for Implementation Research, implementation research, lay antiretroviral therapy adherence counselors, mobile phone

## Abstract

**Background:**

South Africa adopted a universal test and treatment program for HIV infection in 2015. The standard of care that people living with HIV receive consists of 3 sessions of readiness counseling delivered by lay counselors (LCs). In the largest antiretroviral therapy (ART) program worldwide, effective and early HIV and ART education and support are key for ensuring ART adoption, adherence, and retention in care. Having LCs to deliver readiness counseling allows for the wide task-sharing of this critical activity but carries the risks of loss of standardization, incomplete content delivery, and inadequate monitoring and supervision. Systems for ensuring that a minimum standard of readiness counseling is delivered to the growing number of people living with HIV are essential in the care cascade. In resource-constrained, high-burden settings, mobile health (mHealth) apps may potentially offer solutions to these treatment gaps by providing content structure and delivery records.

**Objective:**

This study aims to explore, at a large Cape Town–based nonprofit HIV care organization, the staff’s perceived preimplementation barriers and facilitators of an mHealth intervention (Masivukeni) developed as a structured app for ART readiness counseling.

**Methods:**

Masivukeni is a laptop-based app that incorporates written content, graphics, short video materials, and participant activities. In total, 20 participants were included in this study. To explore how an mHealth intervention might be adopted across different staff levels within the organization, we conducted 7 semistructured interviews (participants: 7/20, 35%) and 3 focus groups (participants in 2 focus groups: 4/20, 20%; participants in 1 focus group: 3/20, 15%) among LCs, supervisors, and their managers. In total, 20 participants were included in this study. Interviews lasted approximately 60 minutes, and focus groups ranged from 90 to 120 minutes. The Consolidated Framework for Implementation Research was used to explore the perceived implementation barriers and facilitators of the Masivukeni mHealth intervention.

**Results:**

Several potential facilitators of Masivukeni were identified. Multimedia and visual elements were generally regarded as aids in content delivery. The interactive learning components were notably helpful, whereas facilitated updates to the adherence curriculum were important to facilitators and managers. The potential to capture administrative information regarding LC delivery and client logging was regarded as an attractive feature. Barriers to implementation included security risks and equipment costs, the high volume of clients to be counseled, and variable computer literacy among LCs. There was uncertainty about the app’s appeal to older clients.

**Conclusions:**

mHealth apps, such as Masivukeni, were perceived as being well placed to address some of the needs of those who deliver ART adherence counseling in South Africa. However, the successful implementation of mHealth apps appeared to be dependent on overcoming certain barriers in this setting.

## Introduction

### Background

In South Africa, the integration of lay counselors (LCs) in HIV health care has revealed some challenges in providing mental health services [1-3]. LCs face many structural challenges in their work, including little space and privacy to conduct counseling and limited support and supervision by health care staff [2,3]. To further compound these challenges, LCs are not part of the South African National Department of Health’s formal employment structure but are rather supervised by their respective facility managers [3]. One of the challenges with integrating LCs in providing antiretroviral therapy (ART) adherence counseling includes the need to standardize content and delivery within a patient-centered framework [4,5]. Current research is focused on ways to optimize the skills of LCs who deliver these interventions [3]. A novel and growing avenue of research to address these challenges is using technology-based interventions [6-9]. Evidence suggests that the use of mobile health (mHealth) apps—mobile and wireless tools used in care settings—can positively impact postintervention health behaviors [10,11].

mHealth-based interventions offer several advantages, such as widespread access to professional care within resource-constrained environments, the incorporation of interactive components to enhance learning and medical education, and a variety of delivery modes (eg, via smartphones, tablets, and laptops) to suit various contexts [9,11]. Another benefit includes visual psychosocial education capabilities that accommodate different learning styles and literacy levels, which is important for lower literacy countries [7]. One such example is Masivukeni, a laptop-based mHealth intervention developed specifically for the South African context for use by LCs who deliver ART adherence counseling [7]. The intervention was informed by social action theory, which emphasizes social relationships and psychoeducation, crucial factors in positive adherence behavior [12]. The Masivukeni intervention provides a scaffold to deliver key information about HIV and ART and activities aimed at addressing behavioral factors that strengthen adherence [12]. Modules are presented in a linear fashion; they can be represented at any time and are delivered in a visual and interactive manner to improve patient interaction and reduce the effect of low literacy levels [7]. Interventions such as Masivukeni offer opportunities to improve access to standardized ART adherence counseling and enhance LCs’ capacity to deliver interventions [13]. However, low-resource health care settings present challenges to wider mHealth implementation because of issues such as limited funding, high costs, and poor infrastructure—factors known to impede the implementation process [10,14].

In South Africa, LCs provide some relief to the health care system but require substantial support, and mHealth solutions may be an effective tool to provide this support [1]. There are approximately 10,000 LCs in South Africa [15]. Although the selection criteria for LCs are highly variable, most LCs have completed secondary schooling [16], and in the Western Cape province, their training is provided by the People Development Center (PDC) [17]. In general, training includes (1) basic information on the HIV/AIDS disease process, (2) an overview of counseling and communication skills, (3) information on legal and ethical issues in HIV testing, (4) conducting pre- and postcounseling processes, and (5) record keeping and reporting practices [17]. Implementing interventions such as Masivukeni may provide standardized counseling, which is essential for ART program retention and patient well-being [2,3,6]. Research in this context is important for future implementation of mHealth apps to address the growing need to standardize content delivery and counseling in low-resource settings.

### Objectives

This study aims to explore staff perceptions of preimplementation barriers and facilitators of mHealth apps using Masivukeni.

## Methods

### Setting and Participants

The study was conducted across 4 sites in Cape Town, South Africa. All 4 sites were operated by TB/HIV Care, one of the largest nonprofit organizations involved in TB (tuberculosis) and HIV treatment and prevention in the Western Cape. TB/HIV Care largely supports local primary health care facilities by providing care to local communities.

Prospective participants were identified as those providing HIV testing services from TB/HIV Care. Participants and key stakeholders from this organization were therefore well placed to offer valuable insights into the perceived preimplementation barriers and facilitators of mHealth interventions, such as Masivukeni. Purposive sampling was used to recruit participants from the following groups: LCs, supervisors (area coordinators), and managers (district coordinators). At each respective site, 3-4 LCs, 1-2 supervisors, and 1-2 managers were recruited. LCs are employed by TB/HIV Care but trained by the Western Cape Government, PDC, previously known as the AIDS Training Information Counseling Center. Participants were required to have at least one year of experience in their respective positions and have no previous exposure to Masivukeni.

A total of 3 focus groups (FGs) (participants in 2 FGs: 4/20, 20%; participants in 1 FG: 3/20, 15%) and 7 semistructured individual interviews (participants: 7/20, 35%) were included in the final analysis to assess perceptions of Masivukeni and mHealth interventions in general. A supervisor was excluded from the analysis because of familiarity with Masivukeni. In total, 16 women and 4 men participated in the study. At each site, we recruited 3-4 LCs, 1-2 supervisors, and 1-2 managers.

Data were collected by university-qualified senior research assistants and overseen by a master’s level project manager. The senior research assistants were trained in qualitative data collection techniques and interviewing skills before data collection. The interviews and FGs were conducted in the participants’ home language (ie, Afrikaans, English, or isiXhosa). The semistructured interviews’ duration was approximately 60 minutes, whereas the duration of the FG discussions was 90 to 120 minutes. All interviews and FGs were audio recorded.

### Approach

The Consolidated Framework for Implementation Research (CFIR) was used to investigate the preliminary *barriers* and *facilitators* of Masivukeni. CFIR is a flexible framework that incorporates constructs and strategies from various implementation theories and offers a collective approach toward implementation research [18-20]. It aims to address implementation at a multilevel system, which includes the intervention’s characteristics, the staff who administer it, and the managers connected to the organization [18]. Determinant frameworks such as CFIR help to address factors hypothesized to influence implementation success [21]; the CFIR consists of 5 domains containing a number of constructs and subconstructs [18,21,22]. Domain 5, which is centered on the implementation process, was not included because this study focused on preimplementation. The interview guides were based on the overall themes of the CFIR domains. Textbox 1 illustrates the 4 domains and an overview of the constructs. *Barriers* are defined as any factors perceived to potentially obstruct the implementation of Masivukeni. *Facilitators* are defined as any factors perceived to potentially enable the implementation of Masivukeni. *Neutral* is defined as a factor perceived as neither obstructing nor enabling the implementation of Masivukeni. To assess potential facilitators and barriers, participants were exposed to a 3-minute video demonstration of Masivukeni [23], followed by a discussion of the app and its potential use in their work environment.

Each domain consists of a set of constructs, whereas domain 3 also contains subconstructs. Domain 1 pertains to the characteristics of the intervention, whereas domains 2 and 3 refer to the inner and outer settings [18]. Domain 4 entails the characteristics of the stakeholders associated with and involved in the intervention [18].

**Domains 1-4 in the Consolidated Framework for Implementation Research.**

Domain 1: Intervention characteristics

- Innovation science
- Evidence strength and quality
- Relative advantage
- Adaptability
- Trialability
- Complexity
- Design quality and packaging
- Cost

Domain 2: Outer setting

- Patient needs and resources
- Cosmopolitanism
- Peer pressure
- External policy and incentives

Domain 3: Inner setting

- Structural characteristics
- Networks and communication
- Culture
- Implementation climate
- Organizational incentives and rewards
- Goals and feedback
- Learning climate
- Readiness for implementation

Domain 4: Characteristics of the individuals

- Knowledge and beliefs about the innovation
- Self-efficacy
- Individual stage of change
- Individual identification with the organization
- Other personal attributes

### Data Management and Analysis

Qualitative data were managed in Nvivo (QSR International, Version 11) and analyzed using the framework method [24] based on the CFIR domains. The framework method is a systematic qualitative analytical technique that produces highly structured outputs of summarized data [24]. For this study, a general deductive approach was applied, as the CFIR contains predefined codes listed as constructs. Constructs deemed significant to the study were determined by the a priori development of the CFIR model. As such, the data were applied to these constructs to determine the feasibility of the intervention. In addition, open-ended coding was included in the analysis to recognize commonalities and peculiarities within the data for the potential development of constructs not listed in the framework. A codebook template from the CFIR database with construct definitions was used [25]. Minor modifications were made to the constructs to fit the context of the study. For example, in domain 4, there is a construct called *other personal attributes*. This construct was broadly defined to include other personal features such as competency, motivation, and values, which can relate to the individuals themselves and to the intervention [25]. For our study’s purpose, this construct was refined to include personally identified challenges, which specified challenges that LCs, supervisors, and managers experienced in their day-to-day work routine.

Two analysts used the same codebook to analyze the data independently. A case report was generated for each FG and individual interviews organized into CFIR domains and constructs. Case reports contained summary statements to illustrate how constructs manifested and were supported by excerpts from raw data sets. Upon completion of analysis, all case reports were compared to identify patterns and differences among the stakeholders. To identify barriers and facilitators, the constructs were subjected to a rating procedure [26]. The rating was based on the valence and strength of the statements under each construct [27]. Valence related to constructs is thought to have a positive or negative influence on implementation. These statements can be positive, negative, neutral, or mixed [27]. A mixed statement contains an equal number of positive and negative statements. In terms of strength, statements may be either strongly (2) or weakly (1) positive or negative. To determine the strength of statements, analysts considered several factors, including the level of agreement between respondents, their use of specific and concrete examples, and choice of language [27]. Any discrepancies in coding were overviewed by a third analyst and discussed with the independent analysts until consensus on the reports was reached.

### Ethical Approval

This study was conducted in accordance with the principles of the Declaration of Helsinki and was approved by the University of Cape Town Human Research Ethics Committee (reference number: 246/2011). All participants in the study participated freely and voluntarily and provided written informed consent.

## Results

### Overview

The following section includes CFIR constructs that were significantly positive or negative among participants during the analysis. Constructs that were not significant to the study or contained no data were excluded. Although open-ended coding for potential inductively developed constructs was included, the analyzed data were easily placed into the predefined constructs and did not necessitate further development of new constructs.

### Domain 1: Intervention Characteristics

#### Facilitator: Perceived Evidence Strength and Quality

Facilitator is a supporting belief that the intervention will achieve desired outcomes. The LCs, supervisors, and managers all felt that Masivukeni would be useful to counselors and their clients. This was especially true when transferring knowledge and facilitating psychosocial education in a standardized manner:

...it’s visual and it’s something that will stick and you will have it right in front of you so you won’t forget information or leave things out that are important because every step is right there...

Manager

#### Facilitator: Relative Advantage

Compared with the current model used in adherence counseling, the LCs, supervisors, and facility managers believed that the mHealth app had several advantages over their existing model, in which flipcharts are used as a supportive visual aid. First, the interactive nature and the visually stimulating components of the activities were thought to be more engaging. Second, all 3 groups of participants felt that this mHealth intervention would be particularly helpful when working with adolescents, especially because they may be attracted to the use of technology. The same advantage for older clientele was not clear, and some LCs mentioned that they would like to keep the books and flipcharts for the *grannies*:

...we thought this is something that sounds like it is going to be more helpful than what we are doing, and we are doing as much as we can with talks, flipcharts and all. But we do notice that the clients, they are different...some they understand well, some they understand with pictures...we thought this is a very good idea because...it is like both of you are involved in this learning activity.

Supervisor

The third major advantage was specific to supervisors and managers. They felt that having an mHealth app may potentially assist with their heavy administrative workload. Currently, Masivukeni is able to record some features of counseling sessions, such as session length. The participants were excited about the sharing and monitoring capabilities of the app, especially the ability to check the number of patients seen per day by each logged-in LC:

...I can easily pull up the stats for the day and see, okay, this particular counsellor has seen these particular clients for this day. … I don’t have to wait for the month end whereby I’m going to get the facility diary and check everything.

Manager

#### Facilitator: Recommendations for Adapting the Intervention to Meet Local Needs

Many recommendations were made to potentially adapt Masivukeni. The LCs felt that the app should have alternate language options available, specifically isiXhosa and Afrikaans—common regional languages in the context of the study. Although counseling sessions are conducted in English, LCs revealed that educational explanations often occurred in the client’s first language, especially if the client was older and less proficient in English. In addition, the participants from each group felt that using an app such as Masivukeni for children, adolescents, adults, and older people was possible but that the content and visual components might need changes for different levels of understanding and appropriateness depending on the age group. The supervisors requested that more information on nutrition and activities aimed at identifying goals and barriers be added to the app. Information on nutrition and identification of barriers were not presented in the demonstration:

...you need to understand also not everybody is perfect in English. You need to understand also there are youngsters, there are “Gogos” [Grandmothers in isiXhosa] of 50 years old. They don’t understand all these things. So, we need to present it in a manner that will suit everyone’s understanding.

Counselor

LCs are required to discuss sensitive topics with patients, such as male circumcision, which can be considered taboo for women to speak about in some cultures. Although LCs are trained in approaching these topics, the app could include activities on sensitive discussions to ease difficulties; this was thought to be potentially helpful.

Other suggested modifications to the intervention included questions regarding its potential electronic capabilities. Specifically, queries centered on whether this type of app could include automated software updates to access the latest adherence curricula. This concern was present across all 3 groups. The participants expressed a need to gain access to changing adherence information and treatment of HIV. Other smaller electronic modifications, such as space for electronic signatures on consent forms, were appealing features. Finally, the groups of participants felt that Masivukeni would be useful as a training tool at the PDC or newly appointed ART LCs in the field, and not just as an aid during ART adherence counseling:

We have to wait for ATICC [PDC] to give us dates so that we can send them [the LCs] for updates...But now, if it can also automatically update the information when there’s something that has been approved so that it can also show up, then it will be also helpful.

Manager

#### Barrier: Complexity to Use the Intervention and Implement It in the Current Setting

There was slight disagreement among the participants in terms of whether the LCs would be able to use the app. Computer literacy among LCs is a concern. The participants revealed that some of this concern was specific to older LCs, who were not familiar with or open to using computers and technology. However, the facility managers felt that using technology would not be a problem, especially if smartphones or tablets were the modes of delivery instead of laptops:

…most of them [are] already using smart phones. It’s only two or three that don’t have smart phones and they already know because a tab[let] is actually similar to a smart phone...They wouldn’t struggle with that at all from my point of view.

Manager

It’s not easy to use computers...even to use my tablet. I do have the tablet but sometimes I’m stuck.

Counselor

#### Neutral: Perceived and Associated Costs

The supervisors and facility managers presented some concern about which entity would, on the one hand, purchase the equipment, and on the other hand, provide its maintenance:

… who’s going to be responsible for the equipment and who is going to buy it?

Supervisor

However, there was some recognition that the maintenance of this app, compared with the upkeep of physical materials such as flipcharts and books, may prove more cost-effective:

I feel it’s easier to change this application than to have all those new posters and things being printed and it tears and it breaks...

Manager

### Domain 2: Outer Setting

#### Facilitator: Perceived Needs and Resources of the Clients

A clear focus on the needs of clients and patients from relevant stakeholders plays a positive role in the effective implementation of an intervention [18,19]. Furthermore, the ability of an intervention to meet these needs is paramount. The clients’ perceived needs centered on access to important information and education on HIV/TB and access to ART adherence education and counseling, which Masivukeni is designed to achieve. The participants felt that addressing barriers to adherence and, in particular, the issue of disclosure was a recurring difficulty among clients. Another important need focused on the issue of accessibility to clinics and facilities. Specifically, clients sometimes struggle to find transport to and from clinics because of financial restraints. When counselors are aware that a client will not be able to return, LCs sometimes combine 2 counseling sessions into 1 session. Thus, the degree of flexibility for each client is exercised by LCs. How Masivukeni addresses issues of accessibility requires further adaptation. Finally, LCs mentioned that clients sometimes migrate to different clinics and facilities around the Western Cape, repeating HIV testing and counseling with various LCs. Masivukeni, as an mHealth app, is currently able to record basic session parameters and may provide a digital space to log information:

Some of the clients, they default because they didn’t disclose at home, and then they give us the wrong addresses...Others, they came, but they work too much, so they don’t have a chance to come to the clinics.

Counselor

When we go to the community, we will find out that [a client]...has got a folder at each and every clinic.

Counselor

#### Neutral: Peer Pressure

Although not a significantly positive or negative construct, there is an awareness of the use of technological interventions in health care in South Africa. Some LCs mentioned MomConnect and asked whether the Masivukeni app was similar to it. The LCs felt that the older LCs and clientele may not respond to technological interventions:

I take it from the MomConnect. There is this web that Government also has, where you connect pregnant mommies onto the site. The only ones that connect to the site is the young ones. You get the 39-year-olds, the 40-year-olds...we want them to connect to the site so that they can see this is danger signs in pregnancy, this is what can go wrong and all of that. But they are not technology orientated.

Counselor

#### Barrier: External Policy and Incentives—Reaching Clients’ Daily Targets

The LCs, supervisors, and facility managers expressed the concern that reaching the required daily targets of clients per day is overwhelming and a significant stressor. Masivukeni contains a structured platform and takes a certain amount of time to complete each activity. There was significant apprehension as to whether the intervention would take more time than currently exercised per client, consequently hindering the daily target attainment:

You have to reach the target; that is why you have to rush the time.

Counselor

### Domain 3: Inner Setting

#### Neutral: Networks and Communications—The Quality and Nature of Relationships

LCs sometimes feel undermined by doctors and nurses. The LCs mentioned that they are often questioned about clients’ whereabouts when the facility is struggling to make contact with clients. A number of factors contribute to difficulties tracking down clients, such as frequent changes in cell phone numbers and client migration. Some LCs reported that this contributed to feelings of stress as they felt responsible for their clients’ whereabouts. However, the relationship between LCs and supervisors was positive. For example, LCs reported that they supported each other during difficult cases.

#### Facilitator: Cultural Values—The Perceived Role of LCs

The role of LCs centered on providing a supportive relationship to their clients, with the aim to remain adherent to ART treatment. Masivukeni, described as an mHealth app used by LCs to facilitate ART adherence [7], may help to foster these relationships and support adherence counseling. Supervisors and managers added that LCs endeavor to empower their clients and help them address barriers to adherence. In this regard, Masivukeni contains specific content to address these barriers [7]. The facility managers agreed that a major aim of the organization is to reach the daily target of clients seen per day, in addition to providing standardized, quality counseling. Although Masivukeni cannot facilitate reaching the daily target, it can facilitate standardized adherence counseling. These organizational values and norms can be broadly aligned with the aims and content of Masivukeni.

#### Facilitator: Implementation Climate as a Shared Receptivity for Change

The LCs, supervisors, and facility managers expressed their openness to the implementation of Masivukeni, although there was some reservation about whether this would interfere with time pressures in their daily duties. This rating reflects the summation of the following subconstructs:

##### Tension for Change

An mHealth solution was needed in the following 3 specific areas: an electronic database to (1) assist with administration and the daily workload; (2) streamline counseling and testing processes; and (3) provide access to evolving medical information and treatments for HIV and ART. A manager stated:

Some facilities have been asking for flip charts now for some time and with the flip charts, things change, then we ask when are we going to update it because the information changes...

##### Compatibility of the Intervention With Existing Systems and Workflow

Differences between LCs and their supervisors and managers were evident in this construct. The LCs and two supervisors seemed concerned that the app would interrupt the workflow of LCs and stop them from completing the daily targets. This is because Masivukeni is highly structured and takes a set amount of time to complete each session. LCs expressed the need to remain flexible for each client’s needs, which can result in different amounts of time spent with their clients. The LCs and a supervisor specifically suggested that this app would be useful at their clubs, where time is not as much of a constraint. However, the facility managers felt that having an app like this for the clubs and individual sessions was useful:

I am thinking this can be used in their groups, clubs. It will be much better to use it in the group. It is –One-on-one might take very long...we can identify the clients that are already having a problem of defaulting or having challenges with their adherence.

Supervisor

LCs also indicated that laptops pose a security risk. Laptops were seen as valuables, and the facilities had been previously burgled. Security, although mentioned, was less of a concern if the app was delivered on a smaller device (such as a smartphone or tablet computer equivalent), which they thought may easily be locked away and kept safe:

...at our clinic, we have got a problem of burglary. They have burgled that container three times. Our chairs like this were gone. The doctor’s computer was gone.

Counselor

##### Relative Priority to Implement

There were some differences between the groups. LCs and many supervisors did not prioritize the implementation of Masivukeni. However, the facility managers expressed that implementing an mHealth app such as Masivukeni, with features that allow for an electronic database, is necessary. A facility manager mentioned that standardization of counseling sessions is paramount to ensure quality service and that if Masivukeni could provide this, it would be important to begin implementation.

##### Learning Climate to Learn a New Method

Despite concerns about time constraints and the apparent rigidity of the app, LCs, supervisors, and facility managers showed a desire to learn to use technology in their field. The participants strongly felt that proper training programs and an ongoing technology support team were necessary for effective implementation:

I think innovation is good. We use technology here now these days. To do something we have got apps, different apps...It will also help the health side if things like this can be developed so that it could be easier...Not only for the user but also for the client.

Counselor

#### Neutral: Readiness for Implementation

There was not enough data for these constructs to be significantly positive or negative. However, 2 subconstructs—leadership engagement and available resources—were manifested among the participants.

##### Leadership Engagement for Future Implementation Endeavors

The supervisors and facility managers expressed that for effective implementation to occur, staff engagement would be necessary, particularly related to training and motivation. Supervisors and facility managers expressed a desire to involve the LCs during the implementation process, collaborate with researchers on further adaptations to the app, and develop implementation strategies that will best fit the organization:

I think if myself, as being the supervisor, is positive about the thing that we try out, then it will be a step in the right direction.

Supervisor

##### Available Resources That Can Include Physical Space and Allotted Time

LCs and supervisors mentioned that there were issues with adequate space management. Specific issues are that LCs share consultation rooms, which they feel compromises confidentiality and privacy during their sessions. Use, safekeeping, and maintenance of the equipment (ie, the Masivukeni laptop) were of some concern for future implementation.

### Domain 4: Individuals Characteristics

#### Facilitator: Knowledge and Beliefs About the Intervention

General sentiments regarding Masivukeni were positive. Participants felt that it may assist LCs by providing counseling and facilitating psychosocial education for clients. However, again, the participants were unsure and concerned about how the structured and step-by-step nature of the app would fit their daily duties. Although not indicated by the researchers or demonstrated in the video, participants projected 2 capabilities onto Masivukeni. The first was electronic database recording for clients that included a sharing feature and internet access to a web-based system.

#### Facilitator: Personally Identified Challenges

LCs mentioned that some difficulties include dealing with an array of complex psychosocial issues, such as HIV-positive pregnant teenagers. The LCs, supervisors, and facility managers indicated that disclosure and clients who default are also significant stressors to LC work. In turn, supervisors and facility managers expressed concerns that LCs are not always prepared with sufficient skills to deal with the variety of difficulties each client presents. Masivukeni may be able to support LCs when dealing with complex psychosocial issues.

## Discussion

### Principal Findings

This study explored the perceived preimplementation barriers and facilitators of an mHealth HIV/ART counseling intervention, Masivukeni, in a South African setting. As technology is used more widely in the public health sphere, particularly in low-resource settings, there is a need to assess effective ways to ensure reliable and sustainable scale-up strategies for mHealth interventions [28-30]. The study revealed general, shared receptivity for the inclusion of technological apps such as Masivukeni in clinics and health care facilities. This shared receptivity is critical for effective implementation [31]. Potential end users of Masivukeni felt that there were several advantages; these entailed (1) the ability to include key counseling techniques and materials to overcome health challenges experienced by patients (such as problem-solving activities), (2) accessibility to subpopulations (such as adolescents) because of the interactive e-environment, (3) the potential for a rapid, real-time update of materials, and (4) the possibility of enhancing the record keeping and monitoring functions required by providers.

LCs, supervisors, and facility managers were also excited about the prospect of using Masivukeni to enhance staff skills. Previous research has shown that LCs trained to use Masivukeni expressed feelings of empowerment [32], which is useful as LCs deal with a variety of complex cases that can cause significant emotional stress [33]. An app that facilitates basic counseling skills and knowledge through interactive and engaging learning can be impactful [11]. Participants felt that Masivukeni would be able to accomplish knowledge transfer and may also help to engage with adolescent subpopulations, who they thought required more attention-grabbing material. There is some evidence suggesting that adolescents and young adults may benefit from tailored, interactive interventions [34]; thus, using mHealth interventions tailored for adolescents may be particularly useful.

Masivukeni can record certain counseling session parameters, such as session length and which psycho-educational topics were covered. It also contains a simple referral system. Adaptations to include a basic electronic record (eRecord) database are possible and should be considered. Initial research on eRecord use in low-resource settings has shown that it may help relieve vast amounts of paperwork [35,36]. eRecord was appealing because it would ease (1) logging client information to help with migration issues and (2) monitoring counseling sessions.

Three key suggestions were noted. First, participants suggested smartphones and tablets would be preferred over computers or laptops as the mode of intervention delivery because LCs are more familiar with that technology, requiring less training. Recent evidence suggests that mobile technology access is on the rise in low-resource settings [28] and may be a better option. In addition, smartphones and tablets, which were perceived as smaller and less costly devices, were also thought to be less of a security threat. The second suggestion entailed the inclusion of several language options, as different languages and various computer literacy levels exist in South Africa [37]. Different literacy levels have been found in other resource-limited settings [37,38]. Training workshops to address computer literacy are needed for successful implementation. Finally, many LCs and supervisors were concerned that Masivukeni’s structured platform might become a significant barrier because of the limited time each LC has with their clients. LCs and supervisors suggested that this app could be used in group therapy wherein time is a significant constraint factor, as opposed to one-on-one sessions. However, if the mHealth app was adapted to fit within each session’s time constraints, this barrier may be less significant. Meeting these adaptations will be important because interventions that pose a perceived threat may evoke resistance to intervention adoption [30].

There are 3 major barriers. The first barrier concerns which entity would be responsible for purchasing costs and maintaining mHealth interventions such as Masivukeni. This is important because a key advantage of using mHealth apps in low-resource settings is their potential to be cost-effective [1,7]. Potential technical support may also become a necessity if staff are not technology-oriented. The second barrier deals with the sentiment that LCs may, at times, feel undermined by other staff members [39]. This study pertained to communication difficulties between doctors and LCs concerning LC roles and responsibilities; this has been reported in previous studies [3,9]. Successful intervention implementation depends on effective communication between staff members [18] and can be negatively affected in low-resource environments because of unclear health system responsibilities [14]. In this case, unclear career paths for LCs and poor communication may play a role in the future success of any intervention implementation, not just mHealth apps. Finally, the LCs revealed that their counseling space was sometimes inadequate and not always private, compromising on confidentiality. Although this barrier may not be specific to Masivukeni, lack of available resources, such as physical space, may hinder the potential implementation of interventions in general [6,7,13]. However, as an example of a tailored mHealth intervention, Masivukeni may be able to fill the need for standardized ART adherence practices [2,3]. Despite these barriers, the implementation climate was open and optimistic.

### Limitations

The demonstration of Masivukeni did not show its latest features and modifications. For example, alternative language options were a concern among the participants. However, later versions of Masivukeni include activities in isiXhosa (the predominant local Bantu language in the Western Cape region). In addition, because the participants were not familiar with the entirety of the intervention and were only exposed to a demonstrational video, the perceptions represented in the study were only an initial indication of the implementation context. However, an investigation into the perceptions of mHealth solutions and existing knowledge around such interventions are informative steps in the direction of intervention adoption and dissemination [21]. Another limitation of this study is the lack of external validity because of the limited number of participants in the sample. However, the in-depth nature of the information collected in this study aimed to address this limitation.

### Conclusions and Future Implications

This study qualitatively explored staff perceptions of preimplementation barriers and facilitators of Masivukeni, an mHealth app. Participants were excited about the potential use of Masivukeni in their workplace and felt that it had several advantages. Opportunities to implement the tool include first including it in the adherence counseling curriculum offered at the local training center. This affords the future counselor the benefit of building the theory and practice of counseling into the platform provided, familiarity with the tool, and mastery over its use, which may lead to less time taken to administer the modules and also ease concerns about lack of skills to use the app. A second implementation recommendation would be to allow the use of the tool in selected clinics in a piecemeal fashion. Hopefully, this would reduce anxieties around the time taken to administer the full intervention and seed out its use without insisting on it in its entirety. These strategies may not only facilitate implementation but also reduce perceived barriers. Participants made various suggestions about the ways in which the app could be adapted, which may show some degree of staff motivation and engagement. However, there were concerns about the compatibility with the LC workflow and load, which may contribute to the app’s poor longevity [40].

Questions regarding the full-scale implementation of apps such as Masivukeni remain. First, where would the app be best suited? LCs and some supervisors felt it would be best suited for group therapy, whereas other supervisors and facility managers felt it would be of best use in individual sessions. Second, how do older LCs and clients, who are not technology-oriented, react to technology use? Can interventions such as Masivukeni be adapted to reach those clients who have transportation challenges? To this end, counseling sessions may be delivered via smartphones or tablets as a possible future adaptation. Future studies should also look at the suitability of mHealth apps such as Masivukeni for adolescents; our study participants felt this app would be particularly useful and engaging for this subpopulation.

## Abbreviations

ART: antiretroviral therapy
CFIR: Consolidated Framework for Implementation Research
FG: focus group
LC: lay counselor
mHealth: mobile health
PDC: People Development Center
TB: tuberculosis
